# Antiviral efficacy of favipiravir against canine distemper virus infection in vitro

**DOI:** 10.1186/s12917-019-2057-8

**Published:** 2019-09-02

**Authors:** Xianghong Xue, Yelei Zhu, Lina Yan, Gary Wong, Peilu Sun, Xuexing Zheng, Xianzhu Xia

**Affiliations:** 1 Department of Virology, School of Public Health, Shandong University, Jinan, 250012 China; 2 Division of Infectious Diseases of Special Animal, Institute of Special Animal and Plant Sciences, The Chinese Academy of Agricultural Sciences, Changchun, 130112 China; 3 Zhejiang Provincial Center for Disease Control and Prevention, Hangzhou, 310051 China; 4 Institute Pasteur of Shanghai, Chinese Academy of Sciences, Shanghai, 200031 China; 5 Département de microbiologie-infectiologie et d′immunologie, Université Laval, QC, Québec G1V 4G2 Canada; 6 Institute of Materia Medical, Shandong Academy of Medical Sciences, Jinan, 250062 China; 7 Institute of Military Veterinary, Academy of Military Medical Sciences, Changchun, 130122 China

**Keywords:** Canine distemper, Canine distemper virus, Favipiravir, Antivirals

## Abstract

**Background:**

Canine distemper (CD) is an acute infectious disease with high morbidity rates caused by a highly contagious pathogen (Canine Morbillivirus, also known as canine distemper virus, CDV). CDV can infect a broad range of carnivores resulting in complex clinical signs. Currently, there is no effective method to treat for CDV infections. Favipiravir (T-705), a pyrazine derivative, was shown to be an effective antiviral drug against RNA viruses, acting on RNA-dependent RNA polymerase (RdRp). However, whether the T-705 has antiviral effects following CDV infection is unclear. Here, we investigated the antiviral effect of T-705 against CDV-3 and CDV-11 strains in Vero and DH82 cell lines.

**Results:**

Our data demonstrated that T-705 significantly inhibited the replication of CDV-3 and CDV-11 in both Vero and DH82 cells at different concentrations, ranging from 2.441 μg/ml to 1250 μg/ml. Additionally, T-705 exhibited efficacious antiviral effects when administered at different time points after virus infection. Cytotoxicity tests showed a slight decline in viability in Vero cells after T-705 treatment, and no apparent cytotoxicity was detected in T-705 treated DH82 cells. Comparison of anti-CDV polyclonal serum only inhibition of CDV in supernatant, T-705 directly inhibited viral replication in cells, and indirectly reduced the amount of virions in supernatant. The combination application of T-705 and anti-CDV polyclonal serum exhibited a rapid and robust inhibition against virions in supernatant and virus replication in cells.

**Conclusions:**

Our data strongly indicated that T-705 effectively inhibited viral replication following CDV infection in vitro, and could be a potential candidate for treatment for CD.

## Background

Canine distemper virus (CDV), a member of the genus Morbillivirus within the family *Paramyxoviridae*, is a highly contagious pathogen that causes a multi-systemic disease with severe immunosuppression in carnivores [1, 2]. CDV infects a broad range of animals, including the *Canidae*, *Procyonidae*, *Felidae*, *Mustelidae*, *Mephitidae*, *Ailuridae*, *Viverridae*, *Hyaenidae* and *Phocidae* [3, 4], causing complex clinical signs including respiratory, gastrointestinal and neurological symptoms. Pathogenic bacterial co-infections are known to complicate the clinical signs of CDV-infected animals [5]. Case-fatality rates of CDV infection ranged from 30 to 80% in most susceptible animals, and up to 100% in ferrets [6–9]. Recently, the natural hosts of CDV were widely expanded, even in non-human primates [6, 10–12]. Rhesus monkeys were found to be naturally infected with CDV in Guangxi Province and Beijing, China, with mortality rates of 5 to 30% [6, 10]. Outbreaks in many endangered species, including the Amur tiger, Ethiopian wolf and giant panda, have also been reported [4, 13–15].

Currently, no antiviral drug has been approved for therapeutic application in wildlife animals against CDV infection. The routine vaccination against CDV has been widely conducted for many years. Modified live vaccines (MLV) have significantly reduced CDV infections in dogs and other carnivores [16]. However, MLV are not completely safe in highly susceptible species [17], and CD outbreaks are known to occur even in vaccinated animals [8].

T-705 (favipiravir; 6-fluoro-3-hydroxy-2-pyrazine carboxamide), developed by Japan Toyama Chemical Industry Co., Ltd., is an antiviral agent with a primary mechanism of suppressing the RNA-dependent RNA polymerase (RdRP) activity. As a purine analog prodrug, T-705 converts to active form T-705 ribofuranosyl-5′-triphosphate (T-705-RTP) in cells, which inhibits viral replication by preventing further extension of the RNA strands [18]. T-705 has been confirmed as an efficient inhibitor against a broad range of RNA viruses with RdRP in vitro and in vivo, such as *influenza virus* [18], *Arena*-[19], *Bunya-*[20, 21], *Flavi-*[22, 23], *Ebola-*[24], *Noro*-[25], and *Paramyxoviridae* [26]. However, the antiviral effect of T-705 on CDV has not yet been investigated.

The genome of CDV is a single-stranded negative-sense RNA, which codes a RdRP protein with a binding domain of ATP and/or purine ribonucleotide triphosphate [27, 28]. Based on the efficacy of T-705 against RNA viruses in previous studies, our presumption is that T-705 may also work on CDV. Thus, in this study, we investigated the inhibitory effect of T-705 against two different CDV strains in Vero and DH82 cells, and compared the inhibitory effects of T-705 with an anti-CDV polyclonal serum. Our findings indicated that T-705 could be a potential anti-CDV drug.

## Results

### Growth characteristics of CDV-3 and CDV-11 in Vero and DH82 cells

**Fig. 1:**
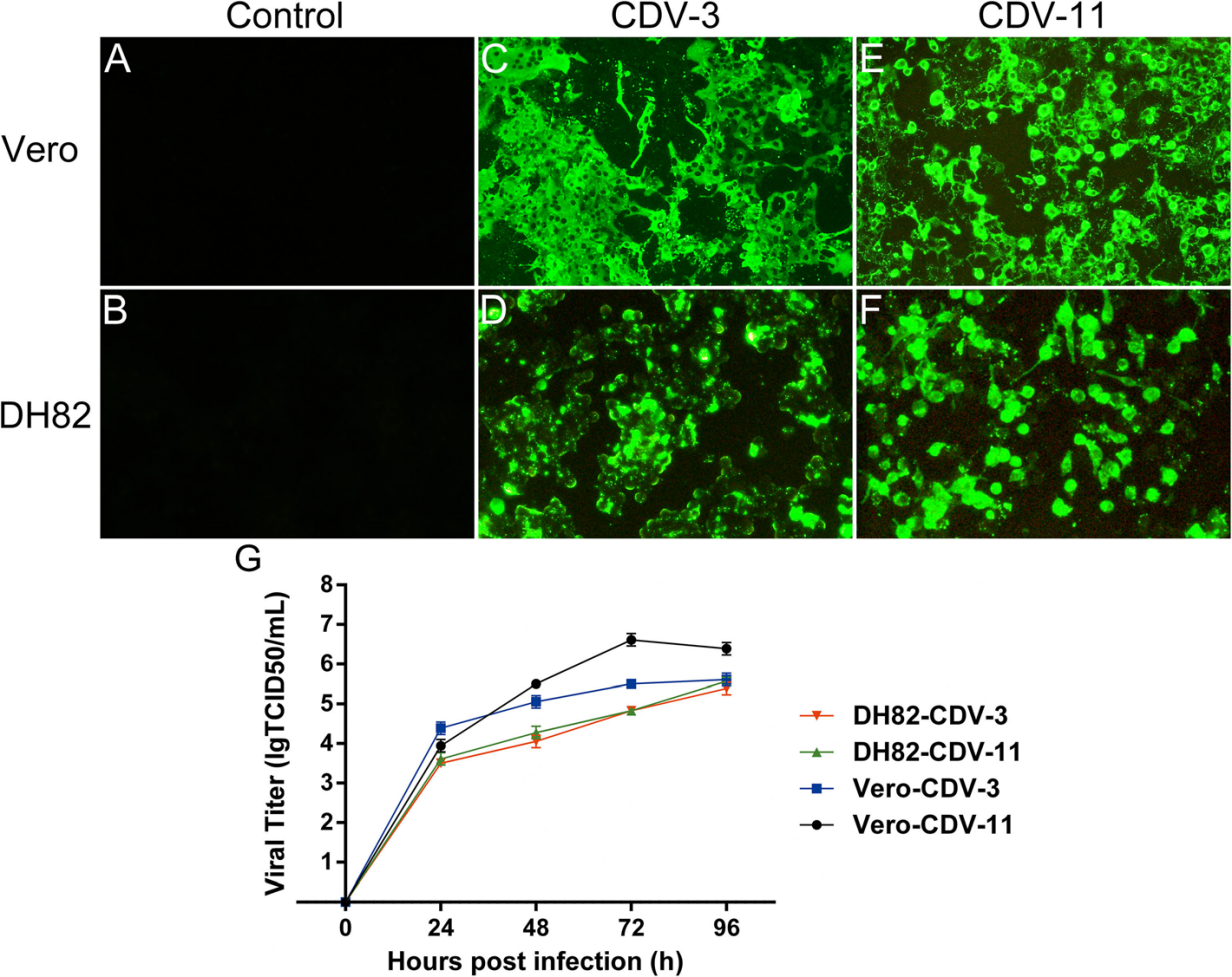
Growth characteristics of CDV-3 and CDV-11 in Vero and DH82 cells. Vero cells were infected with CDV-3 (**c**) and CDV-11 (**e**) at a MOI of 0.1, DH82 cells were infected with CDV-3 (**d**) and CDV-11 (**f**) at a MOI of 0.1, Vero (**a**) and DH82 cells (**b**) with no CDV infection were served as controls. Cells were incubated for 3 days and then fixed and stained with CDV monoclonal antibody specific for nucleoprotein. (Magnification, × 200) (**g**) Growth curves of CDV were performed in Vero and DH82 cells. Cells were infected with CDV-3 or CDV-11 at a MOI of 0.1 and incubated at 37 °C. Viruses were collected at 0, 24, 48, 72, and 96 h p.i. and viral titers tested as described in Materials and Methods. Experiments were both carried out in triplicate

The growth characteristics of CDV-3 and CDV-11 strains in Vero and DH82 cells were determined by indirect immunofluorescence assay (IFA). Both Vero and DH82 cell lines inoculated with CDV-3 or CDV-11, exhibited a strongly positive reaction signal with anti-CDV N monoclonal antibody (Fig. 1c-f); in contrast, no positive reaction signal with anti-CDV N monoclonal antibody was observed in mock cells (Fig. 1a and b. In addition, 50% tissue culture infectious dose per milliliter assay (TCID_50_) was used to determine the viral titers of cultured viruses at different time points (Fig. 1g). In Vero cells, viral titers of CDV-3 and CDV-11 peaked at 10^5.5^ and 10^6.6^ TCID_50_/ml at 72 h, respectively, and maintained a plateau between 72 h and 96 h. In DH82 cells, CDV exhibited a continuous increase of viral titers in the tested range of time points, and viral titers of CDV-3 and CDV-11 both peaked approximately at 10^5.5^ TCID_50_/ml at 96 h.

### Cytotoxicity of T-705

**Fig. 2:**
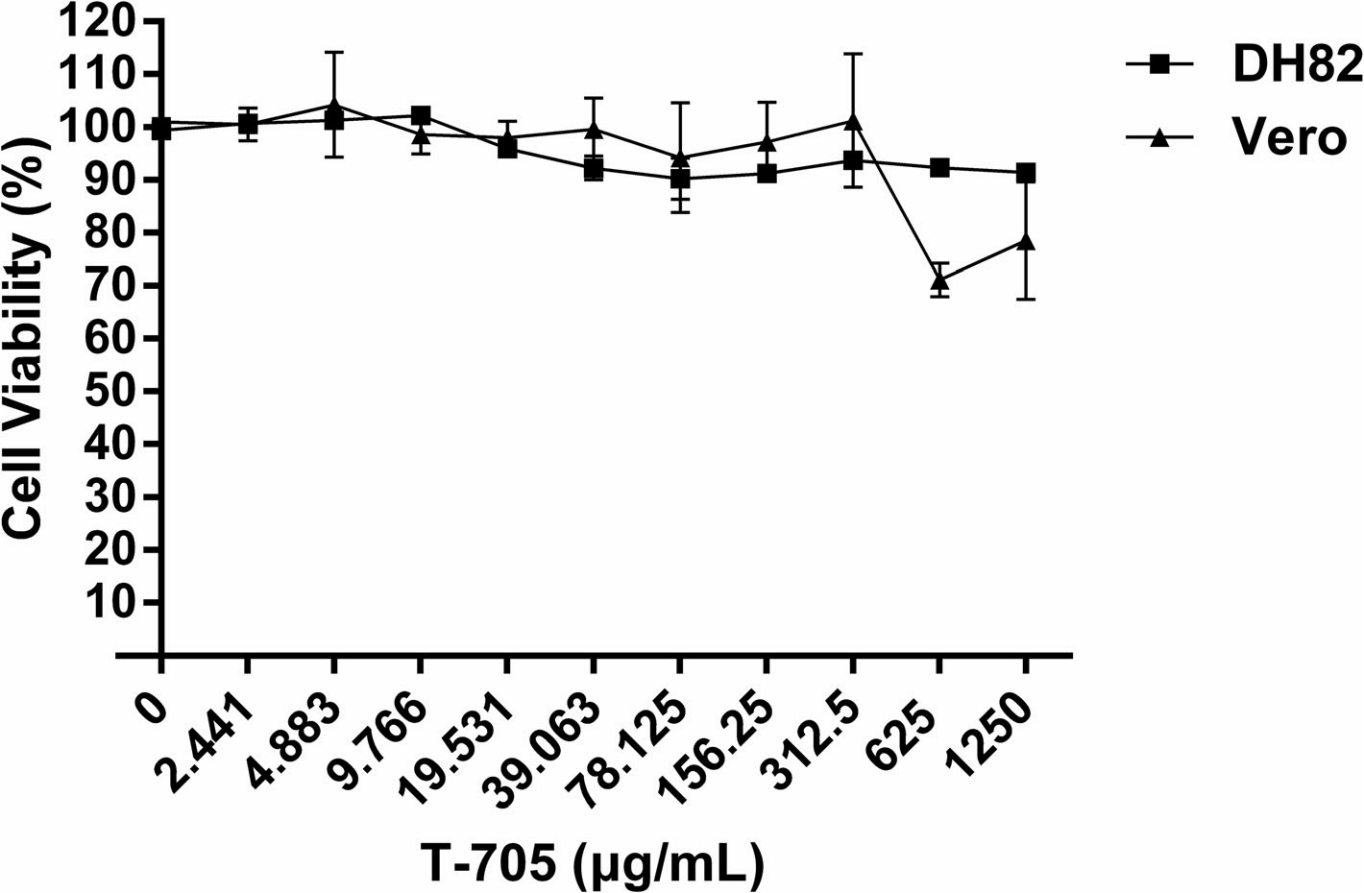
Cytotoxicity test of T-705 in Vero and DH82 cells. Different concentrations of T-705 were added in Vero and DH82 cells. After incubation at 37  °C for 96 h, CCK-8 was added and incubated for 2 h to determine the absorbance at 450 nm. Cells with no drugs were served as controls. Cell viability was measured by comparing the drug-treated cells with controls. Both experiments were carried out in triplicate

The CCK-8 method was performed on Vero and DH82 cells to determine the cytotoxic levels of T-705 (Fig. 2). A slight decline of viability of Vero cells treated with T-705 was observed at concentrations of 625 μg/ml and 1250 μg/ml. No obvious cytotoxicity was observed in T-705 treated DH82 cells for all tested drug concentrations. Moreover, the CC_50_ (50% cytotoxic concentration) values of T-705 in both cell lines were estimated to be higher than 1250 μg/ml.

### Inhibitory effects of T-705 on replication of CDV in Vero and DH82 cell lines

**Fig. 3:**
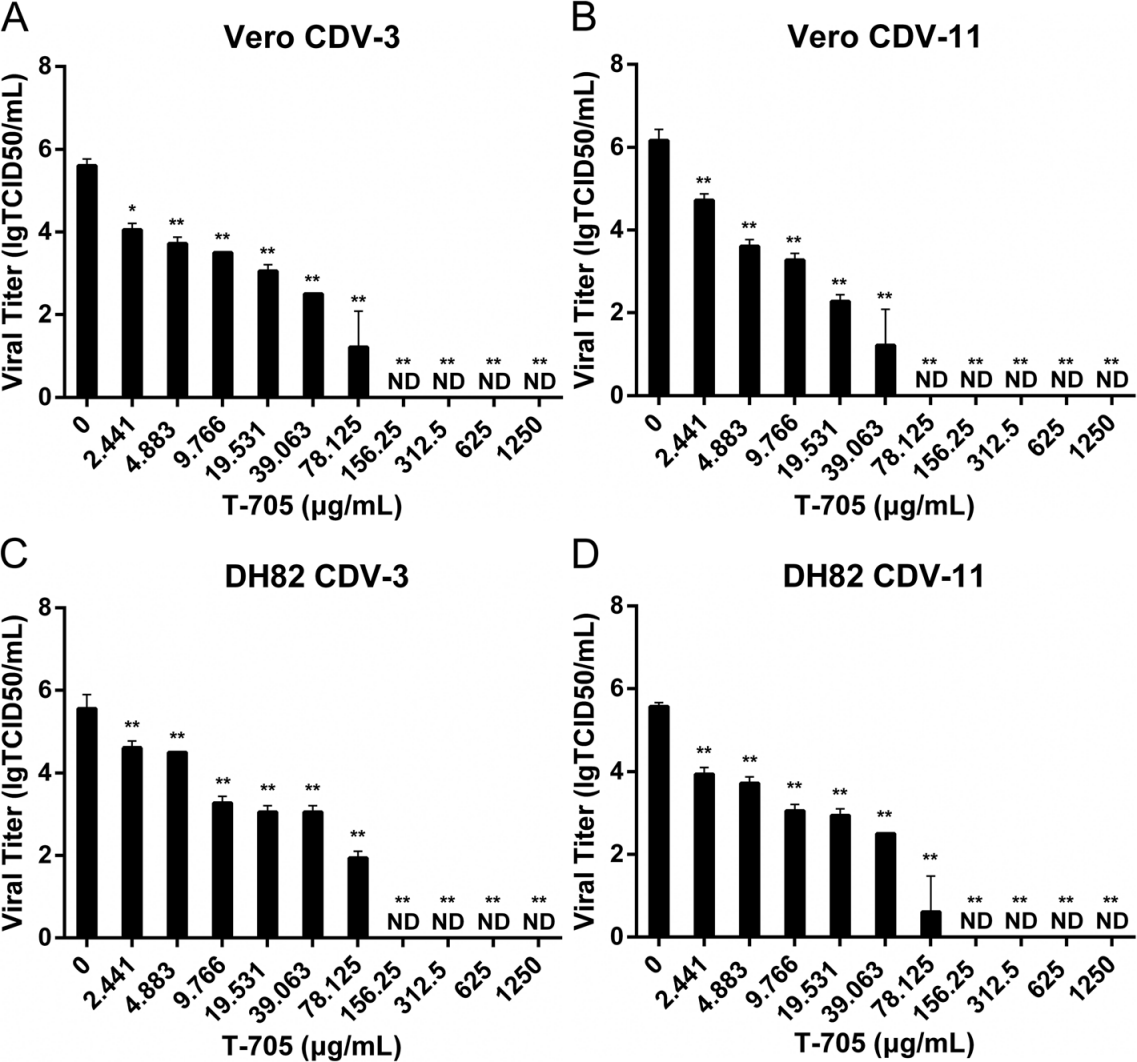
Inhibition of CDV by T-705 in two cell lines. Vero and DH82 cells were infected with CDV-3 (**a** and **c**) and CDV-11 (**b** and **d**) at a MOI of 0.1. Cells were exposed to different compound concentrations at the same time. Cells and the culture medium were collected at 96 h p.i. for virus titration. Cells with no drugs were served as controls. Experiments were carried out in triplicate. Statistical significance was analyzed by one-way ANOVA (Dunnett’s t-test). *, *P* < 0.01; **, *P* < 0.0001. ND means non-detectable

To investigate whether T-705 could inhibit replication of CDV, the antiviral effect of T-705 against CDV-3 and CDV-11 strains was evaluated in both Vero and DH82 cells (Fig. 3). The T-705 was simultaneously added with CDV strains to both cell lines. T-705 significantly reduced viral titers in both cell lines by a dose-dependent manner. Complete suppression of viral replication by approximately 5 or 6 logs was observed with T-705 at concentration levels of 78.125 μg/ml in CDV-11-infected Vero cells, 156.25 μg/ml in CDV-11-infected DH82 cells, and CDV-3-infected Vero and DH82 cells.

To assess the antiviral effects of T-705 against CDV, the half maximal inhibitory concentration (IC_50_) of T-705 against CDV was determined (Table 1). The values of IC_50_ for CDV-3-Vero, CDV-11-Vero, CDV-3-DH82 and CDV-11-DH82 were 25.2 μg/ml (151.2 μM), 7.05 μg/ml (42.3 μM), 33.54 μg/ml (201.24 μM) and 16.97 μg/ml (101 μM), respectively, with selectivity indexes (SI) of higher than 49.6, 177.3 in Vero cells and higher than 37.27 and 73.66 in DH82 cells. Our results indicated that T-705 could significantly inhibit the replication of CDV-3 and CDV-11 in Vero and DH82 cells within the tested range.

**Table 1:** Inhibitory effects of T-705 against CDV replication in different cell lines

Virus	CC_50_ ± SD (μg/ml)^a^		IC_50_ ± SD (μg/ml)^a^		SI ^b^	
	Vero	DH82	Vero	DH82	Vero	DH82
CDV-3	> 1250	> 1250	25.20 ± 2.69	33.54 ± 1.20	> 49.60	> 37.27
CDV-11	*>* 1250	> 1250	7.05 ± 1.87	16.97 *±* 3.35	> 177.30	> 73.66

^a^Determined from 3 independent experiments

^b^Selectivity index (SI) calculated as CC_50_/IC_50_

### T-705 moment-of-addition experiment

**Fig. 4:**
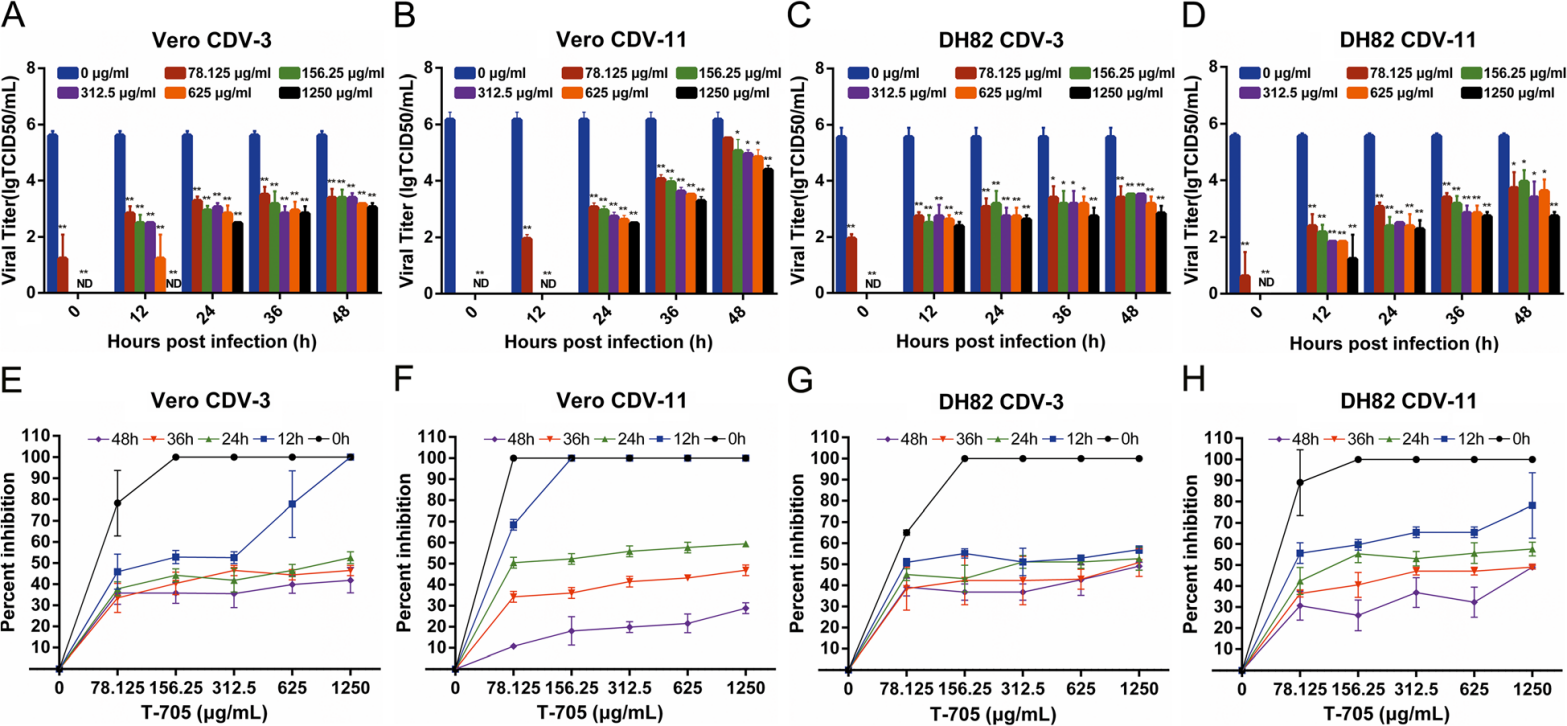
Inhibition of CDV by T-705 at different time points after virus infection and reductions of viral titers by T-705. Cells were infected with both analyzed strains at a MOI of 0.1 and then incubated with different concentrations of T-705 at the indicated time points. Cells and the culture medium were collected at 96 h p.i. to determine viral titers (**a**-**d**) and calculate the inhibition percentages of T-705 (**e**-**h**). Untreated cells were served as controls. Percentages of inhibition were measured by comparing the viral titer of drug-treated to control cells. All experiments were carried out in triplicate. Statistical significance was analyzed by one-way ANOVA (Dunnett’s t-test). *, *P* < 0.01; **, *P* < 0.0001. ND means non-detectable

Moment-of-addition experiments were performed to determine whether T-705 had effective and stable antiviral capacity in CDV-infected Vero and DH82 cells at different moments of drug additions. Inhibition percentages of T-705 were not as 100% as added immediately at 0 h, but still more than 30% in late moment of additions at 12 h to 48 h p.i.. In comparison of the mock-treated but virus-infected cells, viral titers were significantly reduced by 1000 times, when T-705 was added at 12 h to 48 h after viral infection (Fig. 4). Therefore, the T-705 has a strong inhibitory effect on CDV replication when added at 12, 24, 36 or 48 h p.i.. However, in this case, at the same moments of addition of the T-705, different concentrations did not significantly affect the virus titers, and the antiviral activity was similar in all tested concentrations of drug (Fig. 4 e-h). These data indicated that even late intervention of CDV-infected cells with small concentrations of T-705 is still significantly effective.

### Comparison of antiviral effects between T-705 and anti-CDV polyclonal serum alone or in combination

**Fig. 5:**
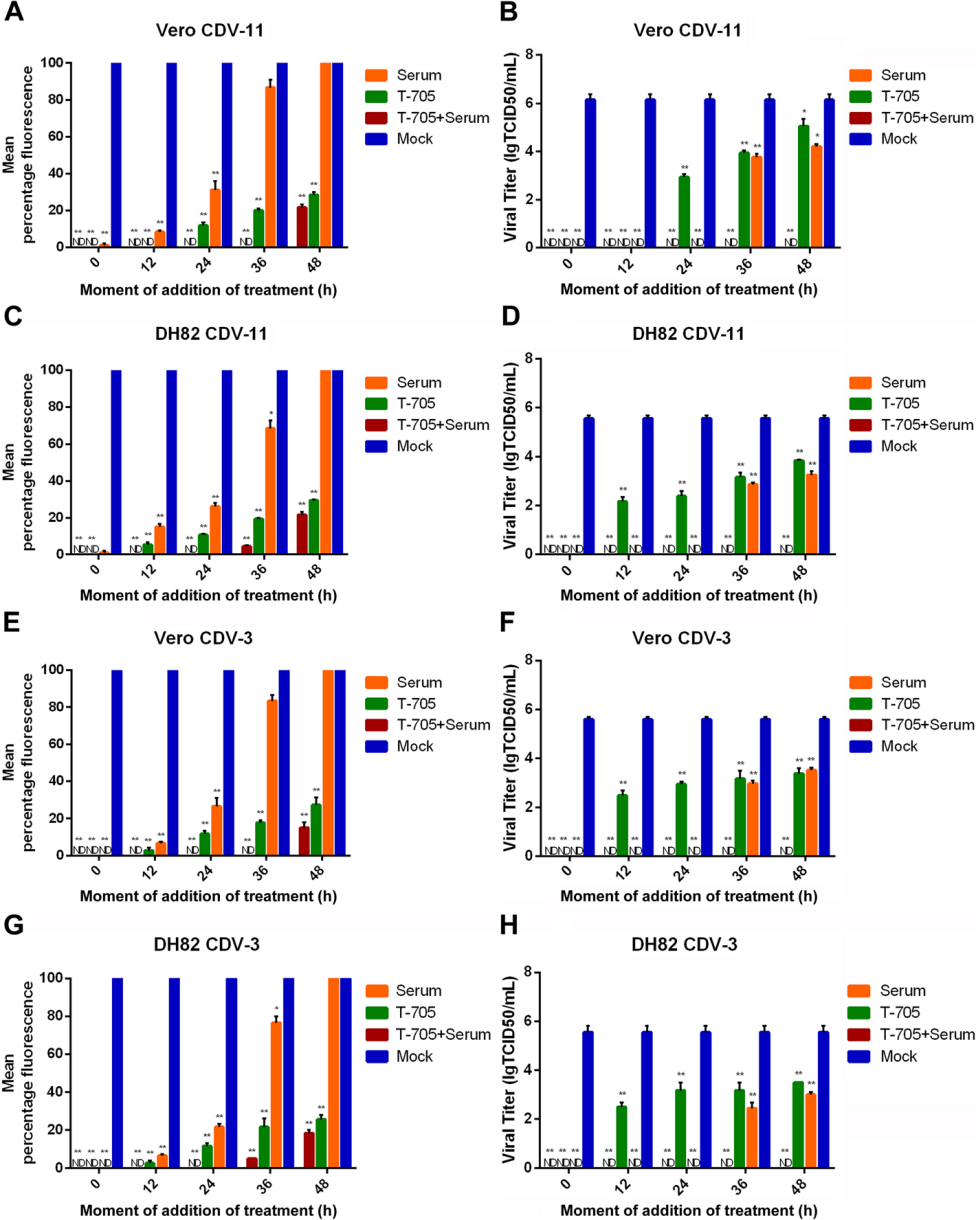
Comparisons of antiviral effects of T-705 and anti-CDV polyclonal serum. The Vero and DH82 cells were treated with T-705 (156.25 μg/ml), anti-CDV polyclonal serum (1:128) and a combination of T-705 (156.25 μg/ml) and anti-CDV polyclonal serum (1:128) at 0, 12, 24, 36 and 48 h p.i. with CDV-3 and CDV-11 at a MOI of 0.1. The mock groups were treated with DMEM p.i. at the same times. The virus titers in supernatant (**b**, **d**, **f**, **h**) were determined and compared with the viral titers in mock group. Viral replications in cells were detected by IFA and the mean fluorescence percentages (**a**, **c**, **e**, **g**) were calculated as the average of fluorescent areas under twenty randomly selected fields. All experiments were carried out in triplicate. Statistical significance was analyzed by one-way ANOVA (Dunnett’s t-test). *, *P* < 0.01; **, *P* < 0.0001

To evaluate the inhibition ability of T-705, we compared the antiviral effects of T-705, anti-CDV polyclonal serum, and a combination of T-705 and anti-CDV polyclonal serum. As shown in Fig. 5a, c, e, g, among the indicated time points, the anti-CDV polyclonal serum had little antiviral efficacy against CDV-3 or CDV-11 in both cells after 36 h p.i., while T-705 showed robust antiviral effects in both cells between 0 and 48 h p.i.. As shown in Fig. 5b, d, f, h, viral titers of both CDV strains in supernatant declined to 0 when CDV-infected Vero or DH82 cells were treated with anti-CDV polyclonal serum or a combination of T-705 and anti-CDV polyclonal serum at 0, 12 and 24 h, while T-705 group showed less significantly hinderance to virions in supernatant. Briefly, anti-CDV polyclonal serum showed a perfect inhibitory effect against CDV virions in supernatant rather than inhibiting viral replication. T-705 exhibited robust antiviral effect in cells where converted to active form. Thus, T-705 combined with anti-CDV polyclonal serum showed an excellent inhibitory effect in supernatant and cells at 48 h p.i. of both CDV strains.

## Discussion

The routine vaccination against CDV MLV has been widely conducted for many years. However, MLV might cause severe diseases and deaths in highly susceptible species [17]. There were many cases of canine distemper outbreaks in vaccinated animals [8, 29]. Cases of dogs suffering encephalitis after vaccination [29] have been reported in young dogs, and the immunity failure is mainly caused by inadequate immune responses to MLV with the presence of maternal antibody in puppy [30, 31]. In addition, whether the MLV is suitable to the endangered species, needs to be considered for the variants with different susceptibilities among wild animals [32]. To address this problem, researchers investigated many antiviral drugs against CDV in vitro*. Carvalho* et al assessed the antiviral activity of several flavonoids and phenolic acids added at different time points [33]. *Lanave* et al assessed the antiviral efficacy of ribavirin and boceprevir alone or in combination against CDV [34]. Other studies showed that caffeic acid has significant anti-CDV effect in Vero cells [35]. *María de Jesús González-Búrquez* et al demonstrated the antiviral activity of the ethanolic extract of Mexican propolis, as well as the synergistic effect that exists between both studied favonoids [36]. Additionally, *Krumm* et al showed that treatment of CDV-infected ferrets, with an inhibitor which targeted the viral polymerase, could reduce viremia and prolong survival [37]. Other therapeutics including neutralizing monoclonal antibodies against the CDV hemagglutinin protein and nucleocapsid protein [38, 39] showed potential immunotherapy against CDV infection in dogs. However, the polyclonal serum has disadvantages of limited sources and heterologous reactions, which restricted its clinical application, especially in wildlife and the endangered species.

There is no effective antiviral molecular currently available to treat for CDV infection in susceptive animals. Thus, T-705, as a confirmed property, inhibits replication of a broad range of RNA viruses both in vitro and in vivo. Here, we have extended the spectrum of T-705, demonstrating robust antiviral activity against CDV in vitro. Our results in this work indicated that T-705 effectively suppressed CDV-3 and CDV-11 in Vero and DH82 cells. In Vero cells, the IC_50_ of T-705 against CDV was 25.2 μg/ml for CDV-3 and 7.05 μg/ml for CDV-11. In DH82 cells, the IC_50_ of T-705 against CDV was 33.54 μg/ml for CDV-3 and 16.97 μg/ml for CDV-11. These findings were comparable to those reported for other RNA viruses, including West Nile virus (IC_50_: 53 μg/ml) [40], foot-and-mouth disease virus (IC_50_: 14 μg/ml) [41], Ebolavirus (IC_50_: 10.5 μg/ml) [24], murine norovirus (IC_50_: 39 μg/ml) [25] and Zika virus (IC_50_:17.4 μg/ml) [42]. D. Jochmans et al demonstrated that recombinant MeV-Edm-GFP was the most sensitive to T-705 treatment of all tested paramyxoviruses, with 90% effective concentration (EC_90_) values of 8.6 μM (1.7 μg/ml), 9.7 μM (1.9 μg/ml), and 13 μM (2.56 μg/ml) for treatment prior to, simultaneous with, and after inoculation [26]. During CDV infection, viral particles are likely to directly attack the peripheral blood mononuclear, macrophages and other immune cells in hosts, and then transmit to bronchial lymph nodes and tonsils within the first 24 h p.i. [43–45]. Finally, it induces a long-term immunosuppression. In this study, T-705 showed robust antiviral effects against CDV in DH82 cells, a cell line from canine macrophages, which suggested that T-705 may be promising as a post-exposure therapeutic drug for CDV infection.

The moment-of-addition experiments proved that T-705 had significant antiviral effects against both CDV strains with addition between 0 and 48 h after virus infection in both cell lines. In previous studies, T-705 was shown to be phosphoribosylated to T-705RTP in cells by intracellular enzymes and then recognized as a purine analog by viral RdRp to inhibit viral replication [26, 46]. That explained why a period of time was needed to reach an effective drug concentration. Virus titers of CDV exhibited a stable decline in DH82 and Vero cells, reaching roughly 10^3.5^ or 10^4^ TCID_50_/ml at 48 h after addition of T-705, in both cell lines, respectively. T-705 still exhibited a more than 30% inhibition rate on replications of CDV-3 or CDV-11 in Vero and DH82 cells even when added at 48 h after virus infection. The antiviral effects of T-705 against CDV showed concentration dependence and negative correlation with additional time. Previous studies demonstrated that the intervention effect of T-705 appeared at an early and intermediate stage of virus replication. In influenza virus, T-705 showed no effect when added at 6 to 10 h after infection [47]. In Tacaribe virus and Junín virus, there was little or no antiviral effect when T-705 was added at 18 h after virus infection [19]. Therefore, it is suspected that T-705 is more effective in combatting CDV infections as a post-exposure treatment. T-705 must have enough time to enter cells and switch to active form, before its antiviral effects exhibit. Comparisons of inhibitory effects between T-705 and anti-CDV polyclonal serum, showed that anti-CDV polyclonal serum only inhibited CDV in supernatant, while T-705 had little hinderance against CDV in supernatant directly. However, T-705 indirectly lessened virions concentration in supernatant by inhibiting viral replication in cells. T-705 combined with anti-CDV polyclonal serum showed an excellent and significant inhibitory effect in both supernatant and cells. Practical applicability of anti-CDV polyclonal serum was restricted by species specificity, but T-705 has a broad spectrum as an antiviral drug. The antiviral effect of T-705 against CDV in vivo was partially restricted for different susceptibility and pathogenicity of CDV in different animal models [48]. Our in vitro results indicated that T-705 was promising as a post-exposure treatment, and the antiviral effect of T-705 in vivo will be evaluated in a future study.

## Conclusions

In this study, our findings demonstrated that T-705 could effectively suppress viral replication in CDV infected Vero and DH82 cells, indicating strong potential of T-705 as a treatment of CDV infections in the future.

## Methods

### Compound, viruses, cells and serum

T-705 (CAS No. 259793–96-9) was purchased from MCE, dissolved in DMEM at a concentration of 5 mg/ml (30 mM), stored at 4 °C and used within 1 week. DH82 cells (ATCC-CRL-10389), a continuous cell line from canine macrophage, and Vero cells (ATCC-81), were both cultured in Dulbecco’s Modified Eagle Medium (DMEM), (Gibco, China) with 10% (v/v) fetal bovine serum (FBS), (Gibco, Australia). CDV-3 was gifted from JiLin Teyan Pharmaceutical Co. Ltd., and CDV-11 was gifted from QiLu Animal Health Products Co. Ltd. Both strains were propagated in Vero and DH82 cells in 2% FBS supplied DMEM, and titrated by 50% endpoint titration. Anti-CDV polyclonal serum was purchased from Changchun Sino Biological Technology Co. Ltd.,

### Cell viability

Viability of Vero and DH82 cells was performed using the Cell Counting Kit-8 (CCK-8, Dojindo, Japan). Briefly, Vero or DH82 cells at 80–90% confluence were grown in a medium containing different concentrations of T-705 in 96-well plates. After incubation at 37 °C for 96 h, 10 μl of CCK-8 solution was added and incubated for a further 2 h at 37 °C. The absorbance at 450 nm was determined. The cell viability was measured by comparing T-705-treated and mock-treated cells. This experiment was carried out in triplication.

### Virus titration

The viral titers of both strains were determined by 50% endpoint titration in Vero cells and expressed as TCID_50_/ml. Vero cells in 96-well plates were combined with serial 10-fold dilutions of the virus, and incubated for 72 h at 37 °C. Cells were fixed with 80% ice-cold acetone. Virus concentrations were measured by IFA. Cells were stained with CDV monoclonal antibody specific to nucleoprotein (VMRD, USA) and Alexa Fluor 488-conjugated Affinipure Goat Anti-Mouse IgG(H + L) (Proteintech, China). The cells were examined by fluorescent microscopy. Mock treated cells were used as a negative control. This experiment was carried out in triplication.

### Viral inhibition assay

To determine the effect of T-705 against CDV in vitro, the culture medium of Vero or DH82 cells infected with CDV-3 or CDV-11 at a MOI of 0.1, were replaced by a medium containing T-705 (0, 78.125, 156.25, 312.5, 625, or 1250 μg/ml) (0,468, 937, 1875, 3750, 7500, 15,000 μM) at the indicated time points. As for time course experiments, five different time points (0 h, 12 h, 24 h, 36 h and 48 h post infection (p.i.)) were set to investigate whether T-705 could still show effective and stable antiviral activity during late administration of drug. In both cases, at 96 h p.i., cells and the culture medium were collected by freezing and thawing three times, and viral titers were measured by IFA as described previously. This experiment was carried out in triplication.

### Comparison of antiviral effects of T-705 and anti-CDV polyclonal serum

The titer of virus neutralizing antibody (VNA) of anti-CDV polyclonal serum was 1:1024. A 250 μl of anti-CDV polyclonal serum was diluted in a 2 ml of DMEM, and the mixture has a final VNA titer of 1:128. The diluted polyclonal sera were tested for inhibition effects in a 6-well plate.

To evaluate the inhibitory effects of T-705 and anti-CDV polyclonal serum, the CDV-3 or CDV-11-infected Vero and DH82 cells were added with T-705 (156.25 μg/ml), anti-CDV polyclonal serum (1:128) and a combination of T-705 (156.25 μg/ml) and anti-CDV polyclonal serum (1:128) at 0, 12, 24, 36 and 48 h, respectively. The mock group was treated with 2% FBS DMEM after infection with CDV-3 or CDV-11. Viral titers in supernatant and viral replication of CDV in cells were determined by IFA at 96 h p.i.. Antiviral effects of tested groups were compared with mock groups treated with 2% FBS DMEM of CDV-infected cells. This experiment was carried out in triplication.

### Statistical analysis

The data were displayed as the mean ± standard deviation (SD). Differences among viral titers were analyzed using GraphPad Prism 6 by one-way ANOVA (Dunnett’s t-test). Statistical significance was considered at * *P* < 0.01 and ** *P* < 0.0001.

## Data Availability

The data used and/or analyzed during the current study available from the corresponding author on reasonable request.

## Abbreviations

CC_50_: 50% cytotoxic concentration
CDV: Canine distemper virus
DMEM: Dulbecco’s modified eagle medium
IC_50_: the half maximal inhibitory concentration
IFA: Immunofluorescence assay
MLV: Modified live vaccines
MOI: Multiply of infection
p.i: post infection
RdRP: RNA-dependent RNA polymerase
SI: Selectivity index
T-705: favipiravir
T-705-RTP: T-705-ribofuranosyl-5′-triphosphate
TCID_50_: 50% tissue culture infectious dose
VNA: Virus neutralizing antibody
